# Antifungal Activity of the Human Uterine Cervical Stem Cells Conditioned Medium (hUCESC-CM) Against *Candida albicans* and Other Medically Relevant Species of *Candida*

**DOI:** 10.3389/fmicb.2018.02818

**Published:** 2018-11-21

**Authors:** José Schneider, Estibaliz Mateo, Cristina Marcos-Arias, Noemi Eiró, Francisco Vizoso, Román Pérez-Fernández, Elena Eraso, Guillermo Quindós

**Affiliations:** ^1^Fundación para la Investigación con Células Madre Uterinas, Gijón, Spain; ^2^Facultad de Ciencias de la Salud, Universidad Rey Juan Carlos, Madrid, Spain; ^3^Laboratorio de Micología Médica, UFI 11/25, Departamento de Inmunología, Microbiología y Parasitología, Facultad de Medicina y Enfermería, Universidad del País Vasco/Euskal Herriko Unibertsitatea (UPV/EHU), Bilbao, Spain; ^4^Unidad de Investigación, Fundación Hospital de Jove, Gijón, Spain; ^5^Departamento de Fisiología-CIMUS, Universidad de Santiago de Compostela, Santiago de Compostela, Spain

**Keywords:** antifungal activity, human uterine cervical stem cells, secretome, *Candida*, *Candida albicans*, *Candida glabrata*, *Candida parapsilosis*

## Abstract

**Background:** Candidiasis is a major cause of human morbidity and mortality. Human uterine cervical stem cells conditioned medium (hUCESC-CM) is obtained from stromal stem cells of the cervical transformation zone, which are in permanent contact with a wide array of potential vaginal pathogens. In previous reports we have found that hUCESC-CM has antitumor and antibacterial potential. Since *Candida* is the most prevalent yeast in the human vagina, it seems plausible that hUCESC-CM might also show activity against it.

**Methods:** In a preliminary step, to evaluate if hUCESC-CM showed any activity at all on *Candida* growth, *in vitro* activities of hUCESC-CM against fluconazole-susceptible reference strains of *Candida albicans, Candida glabrata, Candida krusei*, and *Candida parapsilosis* were studied with a microdilution method on RPMI 1640, using the BioScreen C microbiological incubator. Each measurement was repeated five times. The same methodology was used subsequently on fluconazole-susceptible and fluconazole-resistant *Candida* isolates from blood and vagina of those species corresponding to the reference strains of *Candida* against which activity had been detected in the previous study. Moreover, two fluconazole-resistant clinical isolates of *Candida auris* from blood and urine were also included.

**Findings:** *In vitro* inhibitory activity of hUCESC-CM ranged from 57.5 to 96.6% growth-reduction against fluconazole-susceptible reference strains of *Candida albicans, Candida glabrata*, and *Candida parapsilosis*. hUCESC-CM also reduced the growth of all fluconazole-susceptible tested vaginal isolates by more than 50%. For fluconazole-resistant isolates, growth-reduction was higher than 67% for *Candida albicans*, regardless of its origin (vagina or blood). The isolate of *Candida auris* from urine with a MIC > 128 μ/ml for fluconazole was also significantly inhibited. However, hUCESC-CM was almost inactive against any of the fluconazole-resistant blood isolates of *Candida glabrata, Candida parapsilosis*, and *Candida auris* tested.

**Interpretation:** This is the first report about the growth-inhibiting properties of conditioned medium from human stromal stem cells against different species of *Candida*. Antifungal activity of stromal stem cells depends on their site of origin, being most effective against *Candida* species most prevalent at that particular location. If confirmed in further studies, these findings might result in a completely new therapeutic approach against superficial and invasive candidiasis.

## Introduction

Invasive candidiasis is a major cause of human morbidity and mortality (Quindós, 2014). Moreover, mucosal candidiasis, such as oral and vaginal candidiasis, are very common diseases: more than 75% of all women suffer from vulvovaginal candidiasis at least once in their life and most denture carriers suffer from *Candida*-associated lesions (Singh et al., 2014; Aguin and Sobel, 2015). In 5–10% of women, vaginal candidiasis recurs several times (Weissenbacher et al., 2009). *Candida albicans* is the prevalent etiology of invasive candidiasis. However, other species, such as *Candida glabrata, Candida parapsilosis*, or *Candida krusei*, are the etiology of an increasing number of candidiasis (Pemán et al., 2012; Puig-Asensio et al., 2014) as well as *Candida auris* that more recently has globally emerged as a nosocomial pathogen (Chowdhary et al., 2018; Quindós et al., 2018). These emergent species of *Candida* frequently show a reduced susceptibility to common antifungal drugs used for treating these diseases, such as fluconazole, voriconazole or the echinocandins, anidulafungin, caspofungin or micafungin (Arendrup and Patterson, 2017; Chowdhary et al., 2018). Treatment of candidiasis represents a significant unsolved clinical challenge. New antifungal compounds are required to meet the great clinical challenge posed by invasive and therapy-resistant superficial candidiasis (McCarthy and Walsh, 2017).

We have recently identified and characterized a new strain of human cervical stromal stem cells, which possess antitumoral effects (Eiró et al., 2014). Moreover, the secretome of these cells also exerts antibiotic, anti-inflammatory and reepithelization-enhancing effects (Bermúdez et al., 2015). Our hypothesis is that they constitute a key element in the defense mechanism of the human uterine transformation zone against its biological threats. Indeed, their secretome, contained in their conditioned culture medium (hUCESC-CM), inhibits proliferation above a certain threshold and induces apoptosis of highly proliferating tumor cells (Eiró et al., 2014). At the same time, it enhances reepithelization, and thus proliferation below the safety limit, and shows antibiotic and anti-inflammatory properties in a corneal ulcer animal model (Bermúdez et al., 2015). All this exactly mimics the situation of the cervical transformation zone, which, as has been outlined above, is in a constant process of controlled hyperproliferation and reepithelization (Mitra et al., 2015), within a proinflammatory environment in which bacteria and other microorganisms abound. Our working theory is that stromal stem cells from that particular area have developed, along evolution, some site-specific properties, directed at protecting the remaining cellular environment from external damage through a paracrine mechanism modulating intrinsic defense mechanisms (Schneider et al., 2016). It is already an accepted fact that, although the defining feature of stem cells is that they are undifferentiated and can be redifferentiated into virtually any kind of mature cell, depending on their site of origin, their features are not exactly the same (Vizoso et al., 2017). One of the potentially threatening microorganisms that colonizes the vagina is *Candida*. Usually its growth is kept under control in the homeostatic environment of the vaginal microbiota (Döderlein flora), comprising a number of other microorganisms, most notably lactobacilli. Indeed, the bacterial concentration in the vagina is the highest in the human body, with the exception of the colon. However, when this physiological equilibrium is disrupted, e.g., in the case of antibiotic treatment, yeasts may grow out of control and produce clinical symptoms, and very often huge discomfort to the patient. In up to 20% of instances, the condition will become chronic, despite the restoration of the normal vaginal microbiota. With this in mind, we have tested the potential homeostasis-restoring and antifungal properties of the hUCESC-CM, in order to verify if its antibiotic potential, already demonstrated in the case of bacteria (Eiró et al., 2014), also extends to the next most frequent agent present in the vaginal medium, namely *Candida*.

## Materials and Methods

The study was approved by the Ethics Committee for Regional Clinical Research of Principado de Asturias (Spain, Ethics reference number 100/13) and by the Ethics Committee of the Universidad del País Vasco/Euskal Herriko Unibertsitatea (UPV/EHU, Bilbao, Spain, CEIAB Ethics reference number M30_2015_248).

### Microorganisms

In a first step, four commercially available reference strains (*Candida albicans* ATCC 90028, *Candida glabrata* ATCC 90030, *Candida krusei* ATCC 6258, and *Candida parapsilosis* ATCC 22019) were tested. Following the results obtained with these reference strains, we repeated the experiments on clinical isolates from actual patients conserved at the microorganism bank of Laboratorio de Micología Médica at UPV/EHU (Medical Mycology Laboratory of the University of the Basque Country at Leioa, Bilbao) and on two clinical isolates of *Candida auris* obtained from patients with candidemia from Hospital Universitario y Politécnico La Fe (Valencia, Spain) (Ruiz-Gaitán et al., 2018).

### Compounds

hUCESC-CM was obtained as previously described (Eiró et al., 2014). The medium was maintained lyophilized until its use. The lyophilized powder was resuspended in 2 ml of double distilled water prior to its use, which mimics the original concentration of the conditioned culture medium of hUCESCs.

### *In vitro* Antifungal Activities hUCESC-CM

Antimicrobial *in vitro* activities against *Candida* were tested as follows: yeast inocula were prepared by growing the isolates on Sabouraud dextrose agar plates for 24 h at 37 °C and adjusting to a final concentration between 1 × 10^3^ and 5 × 10^3^ cells/ml in RPMI 1640 medium buffered to pH 7.0 with 0.165 M morpholinepropanesulfonic acid. A 100 μl suspension of each *Candida* strain and 100 μl of the conditioned media assayed (at a final 1:2 dilution) were added to each well of the dedicated 100 well-microtiter plate supplied with the BioScreen System (BioScreen C MBR; LabSystems, Helsinki, Finland). Culture medium and yeast-free controls were included in triplicate in each assay. The first control included 100 μl hUCESC-CM and 100 μl double-distilled water, and the second one, 100 μl inoculum in RPMI and 100 μl double distilled water. The plates were cultivated at 37°C for 24–72 h in a computer-controlled microbiological incubator (BioScreen C MBR; LabSystems, Helsinki, Finland). Antifungal activities of the compounds were studied by means of the optical density measured every hour in the incubator. Each experiment was repeated five times on two consecutive days.

### Yeast Staining for Microscopy

Yeast viability and morphology of three strains, *Candida glabrata* ATCC 90030, *Candida albicans* UPV-15-178 and *Candida albicans* UPV-15-154 were checked after 24 h incubation. One microliter of FUN-1^®^dye from the kit LIVE/DEAD Yeast Viability Kit (Thermo Fisher Scientific S.L., United States) was added both to treated and untreated samples and incubated in the dark for 30 min. The effect of hUCESC-CM on plasma membrane integrity and metabolic function of *Candida* cells was visualized using an Olympus Fluoview FV500 confocal microscope.

### Cytokine Expression in hUCESC-CM

We studied the cytokine content of our study medium by means of a human cytokine antibody array as previously described (Eiró et al., 2014).

### Statistical Analysis

The growth reduction percentage at each time interval was calculated relative to the growth control. Comparisons between group values were performed by means of Student’s *t*-test, *p* < 0.05 being regarded as statistically significant. Comparisons of protein expression in media between groups were performed by means of one-way ANOVA and Bonferroni *post hoc*.

## Results

**FIGURE 1:**
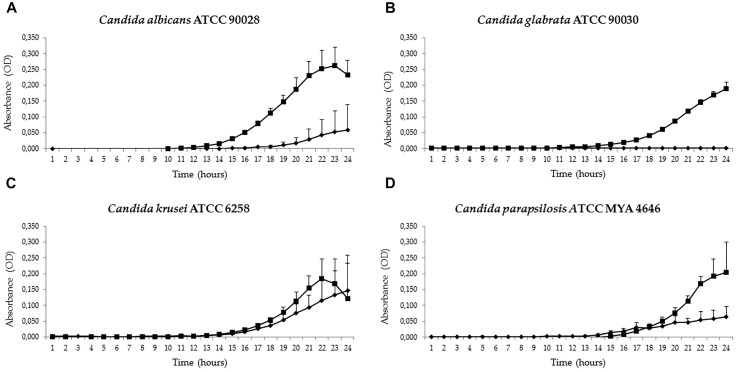
Antifungal activities of conditioned culture medium (hUCESC-CM) against the reference strains *Candida albicans* ATCC 90028 **(A)**, *Candida glabrata* ATCC 90030 **(B)**, *Candida krusei* ATCC 6258 **(C)**, and *Candida parapsilosis* ATCC 22019 **(D)**. Growth curves for the first 24 h of culture without huCESCs-CM (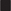) and in presence of huCESCs-CM (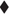).

**FIGURE 2:**
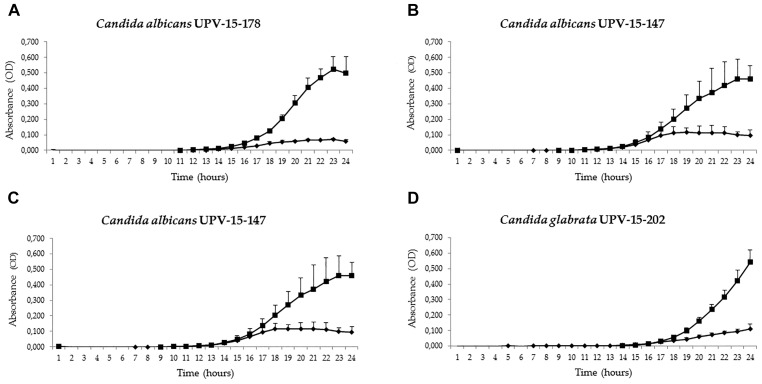
Antifungal activities of conditioned culture medium (hUCESC-CM) against fluconazole-susceptible and fluconazole-resistant clinical isolates of *Candida albicans* and *Candida glabrata* both from blood (UPV-15-178 and UPV-03-282) and vagina (UPV-15-147 and UPV-15-202). Growth curves for the first 24 h of culture without huCESCs-CM (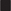) and in presence of huCESCs-CM (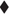).

All the results are summarized in Table 1 and the OD readings for the first 24 h, the period during which the effect of hUCESC-CM was most marked, are represented in Figures 1, 2.

**Table 1:** Twenty four hours *in vitro* activity of human stromal uterine cervical stem cell conditioned medium (hUCESCs-CM) against *Candida.*

*Candida* strains	Origin	Fluconazole susceptibility	Growth inhibition (%)	*p*-value
*C. albicans* ATCC 90028	Blood	Susceptible	57.5	<0.05
*C. glabrata* ATCC 90030	Blood	Susceptible	96.6	<0.05
*C. krusei* ATCC 6258	Sputum	Susceptible	-11.9	0.28
*C. parapsilosis* ATCC MYA 4646	Skin	Susceptible	72.9	<0.05
*C. albicans* UPV-15-178	Blood	Susceptible	88.6	<0.05
*C. albicans* UPV-15-154	Blood	Resistant	67.1	<0.05
*C. albicans* UPV-12-117	Vagina	Susceptible	51.6	<0.05
*C. albicans* UPV-15-147	Vagina	Resistant	79.4	<0.05
*C. glabrata* UPV-03-282	Blood	Susceptible	24.8	<0.05
*C. glabrata* UPV-07-200	Blood	Resistant	0	–
*C. glabrata* UPV-15-141	Vagina	Susceptible	73.1	<0.05
*C. glabrata* UPV-15-202	Vagina	Resistant	81.9	<0.05
*C. parapsilosis* UPV-07-073	Blood	Susceptible	0	–
*C. parapsilosis* UPV-07-058	Blood	Resistant	0	–
*C. parapsilosis* UPV-12-241	Vagina	Susceptible	60.5	<0.05
*C. auris* UPV-17-257	Blood	Resistant	18.3	0.3
*C. auris* UPV-17-285	Urine	Resistant	56	<0.05


In the preliminary study on *Candida* reference strains, antifungal activity, recorded as a reduction of cell growth compared to the control after 24 h of culture at 37°C, was excellent against *Candida glabrata* (96.6%), *Candida parapsilosis* (72.9%), and *Candida albicans* (57.5%). However, there was no clear activity against *Candida krusei*, whose growth seemed even to be slightly enhanced by the presence of hUCESC-CM, although not significantly so (Figure 1 and Table 1). The antifungal activity against *Candida glabrata* of hUCESC-CM was particularly striking (Supplementary Figure S1). When repeating the study on clinical isolates from our microorganism collection, the antifungal activity of hUCESC-CM was equally effective against all fluconazole-susceptible tested isolates (*Candida albicans, Candida glabrata*, and *Candida parapsilosis*) which had been obtained from the vagina of patients. However, for those obtained from the blood of patients, and for fluconazole-resistant isolates, the situation was different: the response was still excellent for *Candida albicans*, regardless of its origin (vagina or blood) and comparable to that of the corresponding fluconazole-susceptible isolates. However, none of the fluconazole-resistant isolates of *Candida glabrata* and *Candida parapsilosis* obtained from the blood of patients showed any response (Figure 2 and Table 1).

Therefore, among the 17 isolates tested, 13 presented growth inhibition in the presence of hUCESC-CM, and in 11 of them, it was higher than 50%. These 11 isolates corresponded to (i) the five *Candida albicans* isolates tested, from blood and vagina (ii) three of the five *Candida glabrata* isolates tested, two from vagina and one from blood (iii) two of the four *Candida parapsilosis* isolates from vagina and skin, and (iv) the *Candida auris* isolate from urine. Moreover, the growth of the five vaginal *Candida* isolates was inhibited in presence of hUCESC-CM (from 51.6 to 81.9%). Moreover, when a 1:4 dilution was tested with three of the clinical isolates, *Candida auris* UPV 17–285, *Candida albicans* UPV 15–147 and *Candida albicans* UPV 15–178, a decrease in growth inhibition of *Candida* was observed (data not shown).

Furthermore, we carried out an exploratory re-analysis on four of the previously tested *Candida albicans* and *Candida glabrata* strains at pH 4.5, the upper limit of physiological vaginal pH. This was prompted by the findings from Danby et al. (2012), who reported different activities of 11 tested antifungal agents against *Candida albicans* and *Candida glabrata* depending on the pH of the culture medium. We found that the activity of hUCESC-CM was largely unchanged against the two susceptible strains of *Candida albicans* and *Candida glabrata* (48.5% vs. 57.5% and 73.0% vs. 73.1% growth inhibition, respectively), and was even enhanced in the only fluconazole-resistant vaginal *Candida albicans* strain tested (97.4% vs. 79.4%). On the other hand, the inhibitory effect was markedly diminished against the fluconazole-resistant *Candida glabrata* strain tested (21.5% vs. 81.9% growth reduction) (Supplementary Figure S2).

**FIGURE 3:**
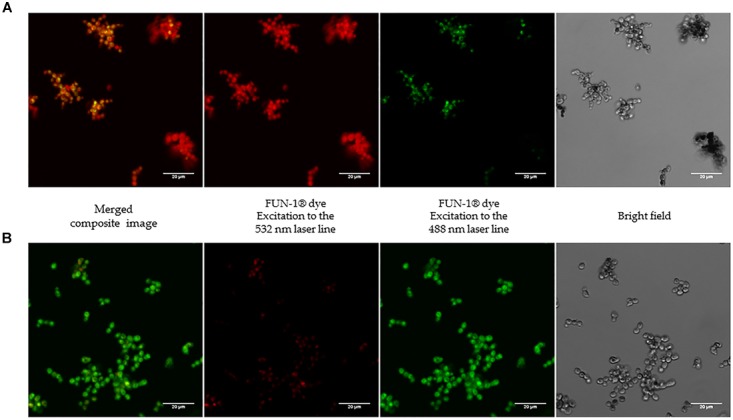
*Candida glabrata* ATCC 90030 viability by Laser Confocal Microscope. Cells stained with FUN-1^®^dye from the kit LIVE/DEAD Yeast Viability Kit after 24 h of incubation without conditioned culture medium (hUCESC-CM) **(A)** and in presence of hUCESC-CM **(B)**.

The effect of hUCESC-CM was easily visualized with the viability assay performed with confocal microscopy (Figure 3). After 24 h of incubation, the yeasts treated with hUCESC-CM appeared green, and thus were not able to convert the yellow–green fluorescent intracellular staining into red-orange, indicating damage in their plasma membrane integrity and metabolic function. However, no variation in cell morphology was observed.

There were no *Candida albicans* germ tubes or hyphae in those wells incubated with hUCESC-CM. However, there were no germ tubes or hyphae present in the wells used as controls (less than 10% *Candida albicans* cells showed any elongation). Some inhibition of *Candida albicans* adhesion and biofilm development was observed in wells with hUCESC-CM. However, these observations are not included, as adhesion and biofilm development or the action of hUCESC-CM against *Candida* preformed biofilms have not been tested in an assay specific for evaluating these actions.

**FIGURE 4:**
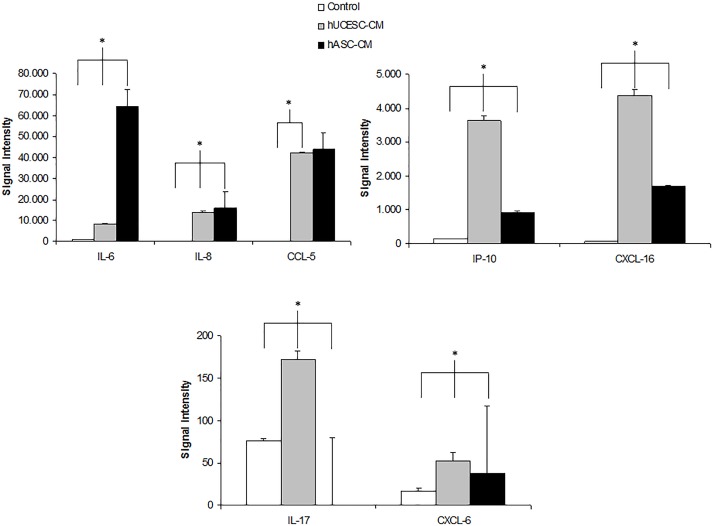
IL-6, IL-8, CCL-5, IP-10, CXC-16, IL-17, and CXCL6 protein expression in culture medium (white bars), in hUCESC-CM (gray bars) and in human adipose stem cells (hASC-CM) (black bars) after 48 h of culture. Error bars represent mean ± SD of signal intensity value as detected in RayBio human cytokine antibody array (^∗^*p* < 0.05).

The study of cytokine expression in our study and control media showed that several cytokines with known antifungal activity (IL-6, IL-8, IL-17, IP-10, CXCL-16, CCL-5, and CCL-6) were expressed at high levels in comparison to those shown by the culture medium itself (the negative control). We also introduced an additional control, namely adipose stem cells (Figure 4), in order to test whether the high cytokine levels expressed by our strain of stem cells (hUCESCs) are peculiar to it, or rather a general feature of stromal stem cells.

## Discussion

To the best of our knowledge, this preliminary report is the first one about the antifungal properties of human stem cell conditioned medium, in this particular case hUCESC-CM. Stem cells are the reservoir from which any other cell type can develop. Therefore, it seems plausible from an evolutionary point of view that they have developed intrinsic defense mechanisms against any potential threats from the environment. Furthermore, it appears that some of those defense mechanisms are more or less developed, depending on the particular site of origin of every particular stem cell population (Schneider et al., 2016). This seems to be corroborated in our study by the finding that the antifungal activity of huCESC-CM was highest against those strains obtained from the vaginal medium, which is the environment in permanent contact with the source of huCESCs, the human cervical transition zone. Conversely, this seems also to indicate that yeasts may modify their phenotype and develop different mechanisms of resistance, depending on the environment in which they thrive, a notion that might have important implications in the clinical setting, when choosing a treatment. In fact, none of the fluconazole-resistant isolates different from *Candida albicans* obtained from the blood of patients was affected in its growth by the incubation with huCESC-CM. On the other hand, we found that the fluconazole-resistant isolate of *Candida albicans* (UPV-15-154) obtained from a patient with systemic candidiasis did respond significantly to hUCESC-CM. This is a highly encouraging finding, since systemic candidiasis is a potentially life-threatening condition, for which the outcome of conventional treatments is often disappointing (Arendrup and Patterson, 2017). Although vaginal candidiasis is not life threatening, on the other hand, it is nevertheless an extremely disturbing condition for the patient, affecting her quality of life. Recurrent vaginal candidiasis, furthermore, is often devoid of a satisfactory treatment. hUCESC-CM showed a significant growth-inhibiting potential against all tested vaginal *Candida* isolates, including against both fluconazole-resistant *Candida albicans* isolates tested. Furthermore, this effect seems to be preserved under pH conditions similar to those found in the vagina, according to our very preliminary sub-analysis, as described in the “Results.” If confirmed in further studies, which are mandatory, this would corroborate the previous findings reported by Danby et al. (2012) in their fundamental study on the variation of antifungal agent activity against *Candida albicans* and *Candida glabrata* under different pH conditions. Considering the known influence of hormones, particularly estrogens, on the vaginal medium and *Candida* growth, it would be also important to study the activity of huCESC-CM against *Candida* in the presence of steroidal hormones mimicking their physiological vaginal levels in premenopausal and postmenopausal women.

In a previous report from our group, we have shown that hUCESC-CM has antibiotic and anti-inflammatory properties (Bermúdez et al., 2015). From the results of the current study, these defense mechanisms against external threats seem to extend also to *Candida*, and in the case of hUCESC-CM, particularly against those strains most prevalent in the vagina, such as *Candida albicans* and *Candida glabrata*. All in all, this may indicate that the transformation zone of the uterine cervix must have developed along evolution a very robust defense mechanism against its multiple biological threats enumerated above.

The mediators of this pleiotropic defense mechanism are largely unknown, although we begin to have some clues. In our previous report about the antitumoral effect of hUCESC-CM conditioned medium (Eiró et al., 2014), we studied its cytokine content by means of a human cytokine antibody array. Several of the cytokines expressed at high levels have known antifungal effects, such as IL-6, IL-8, IL-17, IP-10, CXCL-16, CCL-5, and CCL-6. In particular, IL-17 is essential for mucocutaneous immunity against *Candida albicans* (Ling et al., 2015). Although the standard mechanism of action of cytokines implies immune cell signaling, there is also evidence that cytokines exert a direct effect on fungal cells. Indeed, Zelante et al. (2012) have reported that IL-17 directly binds to *Candida albicans*, hereby impairing its growth independently from the presence of immune system cells. Moreover Li et al. (2013) have reported that IL-17 directly blocks proliferation of other eukaryotic cells, such as neural stem cells, resulting in significantly reduced numbers of astrocytes and oligodendrocyte precursor cells. On a purely speculative level, a similar effect could take place against cells of *Candida*. Thus, in addition to its proinflammatory role through activation of the immune system, IL-17 may have direct effects on *Candida* cell proliferation. As can be seen from Figure 4, IL-17 levels in hUCESC-CM were several times higher than those found either in the negative control (the culture medium) or, interestingly, in another widely studied strain of stem cells which we used as an additional control in this experiment, namely adipose stem cells, indicating that this high level of interleukins with antifungal properties is not a general feature of stromal stem cells, but a specific one of our strain of hUCESCs, most probably due to evolutionary pressure induced by the threat posed by *Candida* in the vagina toward the extremely fragile transformation zone of the cervix (Schneider et al., 2016). It is at present completely unknown if the other cytokines identified by us and expressed at high levels in hUCESC-CM also have a direct antifungal action, like IL-17, besides the “classical” one involving host immune cells, but it might well be that at least some of them do. Besides, they certainly induce an immune antifungal response in the living tissue surrounding the transformation zone of the cervix under physiological conditions in the living organism. Therefore, speculatively, hUCESC-CM, used as a local antifungal treatment in the vagina, might have a dual action: the direct one shown in this study, plus the “classical” one through induction of the host immune cells.

Fluconazole is a first choice antifungal drug in the treatment of mucosal candidiasis, such as *Candida* vulvovaginitis (single oral dose) and one of the first choices in the targeted treatment of invasive candidiasis caused by those species of *Candida* usually susceptible to fluconazole, such as *Candida albicans* and *Candida parapsilosis*. However, superficial and deep candidiasis caused by species less susceptible of even resistant to fluconazole, such as *Candida krusei, Candida glabrata* or *Candida auris*, are becoming more frequent (Arendrup and Patterson, 2017; Chowdhary et al., 2018). Some of these species may be resistant to echinocandins and even amphotericin B. In these important medical challenges, new therapeutic alternatives are increasingly necessary, with either new drugs, combining two or more of the existing ones, finding new indications for old drugs or developing new biological therapies that allow action to be taken on virulence factors, cell differentiation or *Candida* metabolism (McCarthy and Walsh, 2017). In the current study, preliminary results of one of these latter approaches have been reported with the intention of contributing new ideas and data to the essential debate on the treatment of those infections, such as candidiasis, caused by pathogens resistant to conventional drugs and treatments.

## Conclusion

We have reported for the first time the growth-inhibiting properties of human cervical stromal stem cells′ conditioned medium (hUCESC-CM) against different species of *Candida*. If confirmed in further studies, these findings might result in a completely new therapeutic approach against candidiasis.

## Author Contributions

JS and GQ contributed to the conception and design of the study and conducted the search for literature, the analysis of data, the drafting of the manuscript, and the critical revision and final approval of the manuscript. EM and CM-A contributed to the conception and design of the study, the search for literature, the acquisition of laboratory data and analysis of data, the drawing of figures, and the critical revision and final approval of the manuscript. NE contributed to the acquiring of laboratory data, analysis of the data, the drawing of Figure 4, and the critical revision and final approval of the manuscript. FV, RP-F, and EE contributed to the acquisition of laboratory data and analysis of data and the critical revision and final approval of the manuscript.

## Conflict of Interest Statement

GQ has received research grants from Astellas Pharma, Pfizer, Merck Sharp & Dohme, and Scynexis. GQ has served on advisory/consultant boards for Merck, Sharp & Dohme, and Scynexis, and he has received speaker honoraria from Abbvie, Astellas Pharma, Merck Sharp & Dohme, Pfizer, and Scynexis. The remaining authors declare that the research was conducted in the absence of any commercial or financial relationships that could be construed as a potential conflict of interest.

## Supplementary Material

FIGURE S1 Antifungal activity of conditioned culture medium (hUCESC-CM) against *Candida glabrata* ATCC 90030. Growth curves for 70 h without huCESCs-CM () and in presence of huCESCs-CM ().

FIGURE S2 Antifungal activities of conditioned culture medium (hUCESC-CM) against fluconazole-susceptible and fluconazole-resistant *Candida albicans* (ATCC 90028 and UPV-15-147) **(a,b)** and *Candida glabrata* (UPV-15-141 and UPV-15-202) **(b,c)**. Growth curves for the first 24 h of culture at pH 4.5 without huCESCs-CM () and in presence of huCESCs-CM ().
