# Corrigendum: Antifungal Activity of the Human Uterine Cervical Stem Cells Conditioned Medium (hUCESC-CM) Against *Candida albicans* and Other Medically Relevant Species of *Candida*

**DOI:** 10.3389/fmicb.2019.01297

**Published:** 2019-06-11

**Authors:** José Schneider, Estibaliz Mateo, Cristina Marcos-Arias, Noemi Eiró, Francisco Vizoso, Román Pérez-Fernández, Elena Eraso, Guillermo Quindós

**Affiliations:** ^1^Fundación para la Investigación con Células Madre Uterinas, Gijón, Spain; ^2^Facultad de Ciencias de la Salud, Universidad Rey Juan Carlos, Madrid, Spain; ^3^Laboratorio de Micología Médica, UFI 11/25, Departamento de Inmunología, Microbiología y Parasitología, Facultad de Medicina y Enfermería, Universidad del País Vasco/Euskal Herriko Unibertsitatea (UPV/EHU), Bilbao, Spain; ^4^Unidad de Investigación, Fundación Hospital de Jove, Gijón, Spain; ^5^Departamento de Fisiología-CIMUS, Universidad de Santiago de Compostela, Santiago de Compostela, Spain

**Keywords:** antifungal activity, human uterine cervical stem cells, secretome, *Candida*, *Candida albicans*, *Candida glabrata*, *Candida parapsilosis*

In the original article, there was a mistake in Figure 2 as published. In Figures 2B,C, we accidently inserted the same image. The *Candida albicans* UPV-15-147 growth curve was therefore reported twice, while the *Candida glabrata* UPV-03-282 growth curve was excluded from the figure entirely. The corrected Figure 2 appears below.

**Figure 2:**
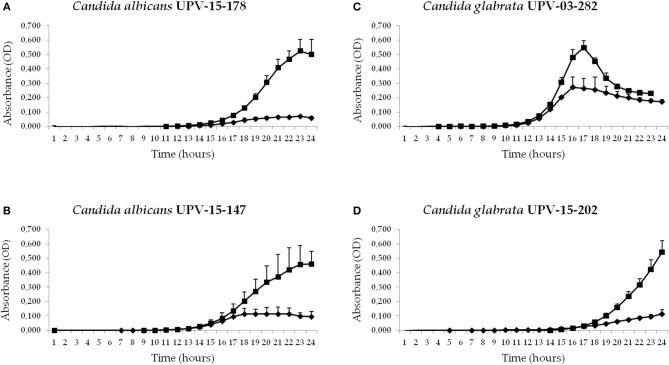
Antifungal activities of conditioned culture medium (hUCESC-CM) against fluconazole-susceptible and fluconazole-resistant clinical isolates of *Candida albicans* and *Candida glabrata* both from blood [UPV-15-178 **(A)** and UPV-03-282 **(B)**] and vagina [UPV-15-147 **(C)** and UPV-15-202 **(D)**]. Growth curves for the first 24 h of culture without huCESCs-CM (■) and in presence of huCESCs-CM (♦).

The authors apologize for this error and state that this does not change the scientific conclusions of the article in any way.

