# Supplementary figures and images for: Antifungal Activity of the Human Uterine Cervical Stem Cells Conditioned Medium (hUCESC-CM) Against Candida albicans and Other Medically Relevant Species of Candida
Source: Front Microbiol. 2018 Nov 21;9:2818. doi: 10.3389/fmicb.2018.02818 (PMC6258777; doi:10.3389/fmicb.2018.02818)

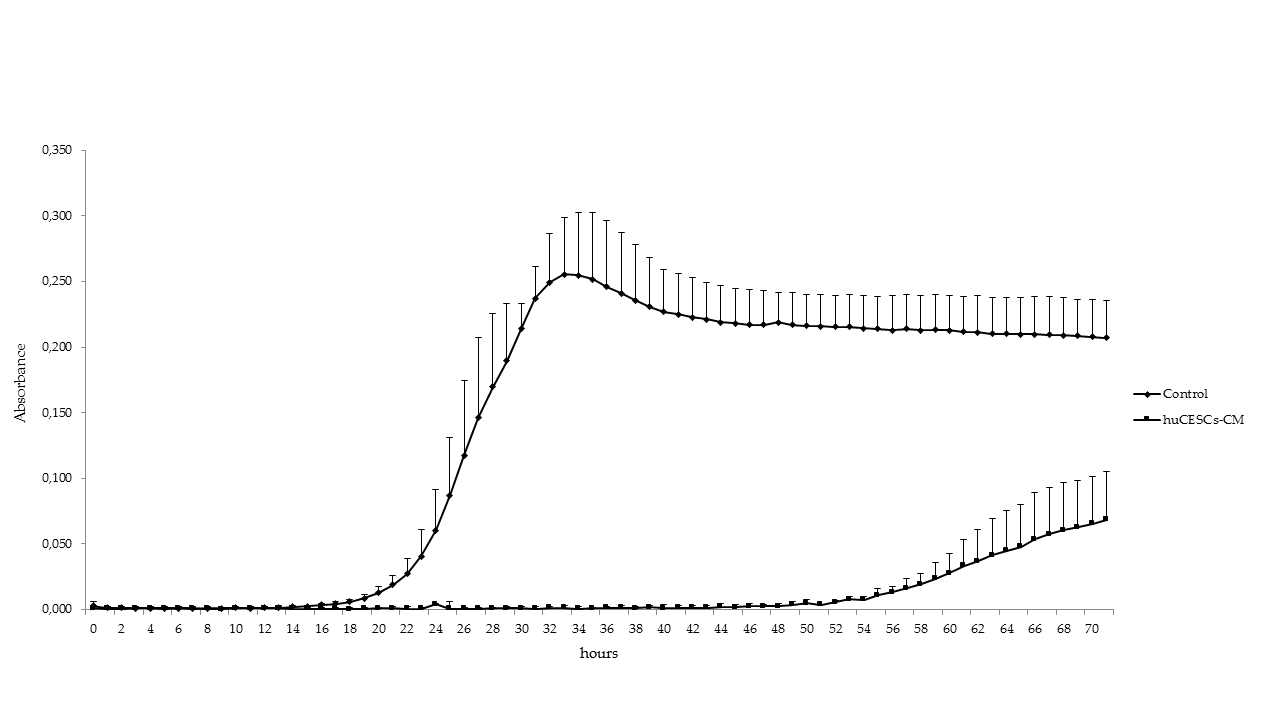

Supplement: FIGURE S1 — Antifungal activity of conditioned culture medium (hUCESC-CM) against Candida glabrata ATCC 90030. Growth curves for 70 h without huCESCs-CM () and in presence of huCESCs-CM (). [file Image_1.TIF]

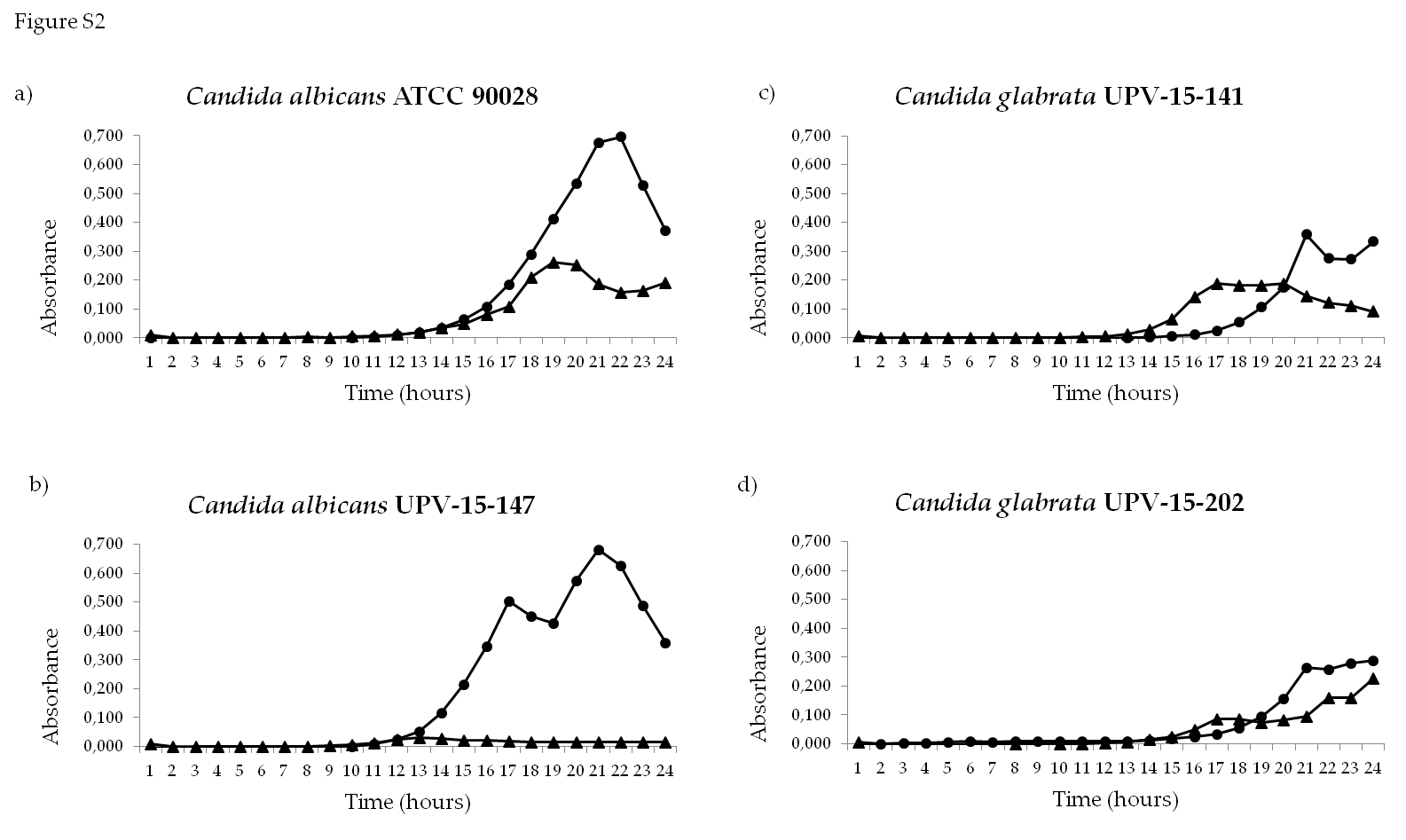

Supplement: FIGURE S2 — Antifungal activities of conditioned culture medium (hUCESC-CM) against fluconazole-susceptible and fluconazole-resistant Candida albicans (ATCC 90028 and UPV-15-147) (a,b) and Candida glabrata (UPV-15-141 and UPV-15-202) (b,c). Growth curves for the first 24 h of culture at pH 4.5 without huCESCs-CM () and in presence of huCESCs-CM (). [file Image_2.TIF]
